# Medical Society of the County of Erie

**Published:** 1911-02

**Authors:** Franklin C. Gram


### SOCIETY PROCEEDINGS

### Medical Society of the County of Erie

REPORTED BY FRANKLIN C. GRAM, M.D., Secretary.

THE eighty-ninth annual meeting of the Medical Society of the County of Erie was held in the rooms of the Society of Natural Sciences, Buffalo Library Building, Monday, December 19, 1910. The meeting was called to order at 8 p. m. by the president, Dr. Grover W. Wende.

On motion, the president appointed Drs. Bennett, Hendee and Trotter, a committee on nominations.

On motion of Dr. Wall, the minutes of the October meeting were adopted as published in the BUFFALO MEDICAL JOURNAL. On motion of Dr. Wall, the proposed amendments to the by-laws were tabled until the next meeting.

The secretary then read the minutes of the council meetings of December 5 and December 12, 1910. On motion of Dr. Wall, the minutes of the council were adopted with the exception of the portions referring to the by-laws, which, under a previous motion, had been tabled.

Dr. T. H. McKee, chairman of the committee on membership, presented a list of twenty-six applicants for membership as follows: Jacob B. Young, 320 East Street; Charles W. Eustace, 101 Seymour Street; Walter R. Ashe, Lake Avenue, Blasdell, N. Y.; James J. Bowen, 2054 Seneca Street; Francis C. Goldsborough, 459 Franklin Street; Bernard F. Schreiner, Buffalo General Hospital; Rudolf C. Miller, 6 North Pearl Street; L. Franklin Anderson, 125 Jefferson Street; William W. Quinton, 232 Elmwood Avenue; Garra K. Lester, Blasdell, N. Y.; John W. Kales, 139 South Division Street; D. F. White, 287 Lafayette Avenue; George W. Gorrill, Buffalo State Hospital; J. P. Barr, 1366 Broadway; Harvey W. Bodamer, 343 Eagle Street; John F. Ryan, 2520 Main Street; George W. T. Lewis, 176 Summit Avenue; Benjamin Jacobson, 383 Sycamore Street; Charles Weil, 561 Genesee Street; Chester T. Stewart, 739 Elmwood Avenue; Albert W. Palmer, 1569 Seneca Street; Chalmers N. Kendrick, 2362 Main Street; Percy C. Cripps, 162 Glenwood Avenue; Charles Battaglia, 126 Dante Place; Leon S. Kurek, 577 Fillmore Avenue; Jesse G. Levy, 241 Cedar Street.

On motion of Dr. Hopkins, the secretary was instructed to cast the ballot of the society for the several applicants and they were thereupon declared duly elected.

Th president announced that this made a total of 166 new members elected during the year 1910.

On motion, the thanks of the society were extended to the committee on membership for its very efficient work.

Dr. Bennett then presented the following nominations of officers for the ensuing year:

For president, Bernard Cohen.
For vice-president, Thomas H. McKee.
For second vice-president, Frank A. Helwig.
For secretary, Franklin C. Gram.
For treasurer, Albert T. Lytle.
For censors, John H. Grant, George L. Brown, Charles Battaglia, Francis E. Fronczak, and Arthur G. Bennett.
For chairman, committee on legislation, F. Park Lewis.
For chairman, committee on membership, Charles A. Wall.
For chairman, committee on public health, Henry R. Hopkins.

For delegates to the Medical Society of the State of New York —1911-1912—F. C. Busch, Edward Clark, George J. Eckel, John H. Pryor, and Eli Long.

The president called for other nominations besides those presented. Daniel V. McClure's name was presented as nominee for president. Lawrence Hendee's name was added to the nominees for censor. There being no further nominations, the president declared the nominations closed, and appointed Drs. Francis and Bethune as tellers. The president instructed the tellers to distribute ballots and collect them, the polls to remain open until every member present had an opportunity to vote.

The president then called vice-president Cohen to the chair and read his annual address.

On motion of Dr. Wall the thanks of the society were tendered to President Wende and his address was ordered to be printed in the BUFFALO MEDICAL JOURNAL.

The secretary's reports having been published after each meeting no report from him was given.

Treasurer Lytle then read his report which was, on motion, adopted and the recommendations contained therein approved. The report is as follows:

December 19, 1910.

*To the Medical Society of the County of Erie*:

In presenting this as the annual report of the treasurer I respectfully call your attention to the fact that the fiscal year of the society ends with December 31, and includes the financial transactions of this meeting and of the next eleven days; therefore, the true annual report of the treasurer will be handed to the auditor so that the auditing committee may report thereon at the first regular meeting of 1911.

Again, the society is to be congratulated upon the fact that the dues of all the members are practically paid up. Of the total number of names reported to be dropped from the roll December 31, only one member known to be residing in Erie County will be dropped for non-payment of dues.

I would respectfully suggest that the treasurer be instructed to create a special account to be known as the permanent or sinking fund, into which shall be transferred at the close of the fiscal year all money in excess of $100 and not otherwise provided for, that remains in the general funds of the society after all proper expenses incurred by the society have been paid; that he be instructed to prepare and present to the society an addition to the by-laws creating and defining the uses and care of such funds; that he be ordered to begin to set aside such money as above described at the close of the fiscal year 1910, and that he be ordered to deposit such funds in such savings banks and trust companies as shall be selected by the council for that purpose.

Respectfully submitted,

ALBERT T. LYTLE,
*Treasurer.*

MEMBERSHIP STATEMENT, DECEMBER 19, 1910.

| | | | |
|---|---|---|---|
| Numbered receipts used to date | | 536 | |
| One member resigning and being reinstated during the year used two receipts | | 1 | |
| Membership roll, December 19, 1910 | | —— | 535 |
| Paying members | | 530 | |
| Honorary members | | 5 | 535 |
| | | —— | —— |
| In good standing December 31, 1909 | | 404 | |
| Additions to date | | 132 | |
| Reinstatements | 24 | | |
| New | 108 | | |
| Total to be accounted for | | —— | 536 |
| Prepaid 1910 dues and assessments (reported in last annual report) | | 1 | |
| Paid by resolution Society | | 51 | |
| Unpaid from all causes, December 19,1910 | | 18 | |
| Paid 1910 dues and assessments, December 19, 1910 | | 466 | |
| | | —— | 536 |
| Treasurer charges himself with 1910 D. and A. from | | 466 | |
| Treasurer charges himself with 1911 D. and A. from | | 1 | |
| On December 19, 1910, with 467 + $5 = | $2,335.00 | —— | |
| Dropped by death | | 4 | |
| Dropped by resignation | | 2 | |
| Dropped by notice of removal and transferred | | 4 | |
| Unpaid December 19, 1910, left city, address lost | | 6 | |

| | | |
|---|---|---|
| Unpaid December 19, 1910, serious illness | 1 | |
| Unpaid December 19, 1910, intentional—"sore" | 1 | |
| Unpaid December 19, 1910, negligent | 4 | |
| (Will pay before December 31, 1910.) | | |
| .. Total unpaid | —— | 22 |
| In good standing December 19, '10: Honorary, 3; Paying 511 | | 514 |
| | | —— |
| | | 536 |

### CASH ACCOUNT.

| | | | |
|---|---|---|---|
| Balance, December 31, 1909 | | | $ 233.41 |
| *Receipts to Dec.* 19, 1910— | | | |
| State assessment, 1910 | | 1,398.00 | |
| County dues, 1910 | | 932.00 | |
| Total, 1910 | $2,330.00 | ——— | |
| State assessment, 1911 | | 3.00 | |
| County dues, 1911 | | 2.00 | |
| Total, 1911 | $5.00 | ——— | |
| Total dues and assessments | $2,335.00 | ——— | |
| Refund board of censors | | 50.00 | |
| Court fines account | | 400.00 | |
| Total board of censors | $450.00 | ——— | |
| Jacobi fund | | 55.00 | |
| Outing fund | | .15 | |
| Total | | ——— | 2,840.15 |
| | | | ——— |
| Total on hand and received | | ——— | 3,073.56 |

### EXPENDITURES TO DECEMBER 19, 1910.

| | | |
|---|---|---|
| M. S. S. N. Y. assessments | $1,218.00 | $ |
| Secretary's office | 204.74 | |
| Treasurer's office | 63.34 | |
| Board of censors | 300.00 | |
| Membership committee | 63.96 | |
| Legislative committee | 3.44 | |
| Public health committee | | |
| Entertainment committee | 168.25 | |
| Contagious hospital committee | 9.20 | |
| Necrology committee | 2.25 | |
| A. M. A. meeting committee | 78.81 | |
| Division of fees committee | 21.25 | |
| Jacobi fund committee | 77.05 | |
| Sundry items | 52.00 | |
| Total expenditures | ——— | $2,252.29 |
| Balance in bank | | 736.27 |
| On hand | | 85.00 |
| State society | 183.00 | |
| Fines account | 150.00 | |
| Outing fund | 18.87 | |
| General fund | 469.40 | |
| Total | ——— | $3,073.66 |

Moved by Dr. Wall that an honorarium of one hundred dollars ($100) each be given to the secretary and to the treasurer for services performed for the society. Carried.

Dr. John H. Grant, chairman of the board of censors, read his annual report which was adopted as follows:

BUFFALO, December 19, 1910.

*To the Medical Society of the County of Erie*:

Details from January 1 to October 24, 1910, are given in periodical reports heretofore submitted. Continuing, on October 25 the Dr. Stevens-Linn Museum complaint was further adjourned to November 10, and to November 22, on which date Stevens, Dr. Hancock, and Herbert M. Steller, claiming to be a clerk employed by Stevens were all discharged by Judge Nash, because, in his opinion, the evidence was not sufficient to hold. Three of our principal witnesses, Pinkerton detectives, failed to appear, also Dr. Linn, and as a result the case fell to the ground although we did the best with what evidence we could produce to prove that Dr. Stevens's Buffalo medical clinic and Linn's museum were in fact a medical advertising company as coming under the Court of Appeals decision, People vs. Woodbury Dermatological Institute, 192, N. Y., 454, that any person not registered as a physician who shall advertise to practise medicine shall be guilty of a misdemeanor.

October 31, 1910, complaints having been made against one Mrs. (Dr.) Davis, of 246 Elk Street, Buffalo, she was arrested for unlawfully practising medicine. Evidence heard before Judge Brennan, November 5, and although we made out a prima facie case from very unwilling witnesses, Mrs. Davis was discharged, yet it is believed this arrest and arraignment will have a deterrent effect.

November 22, 1910, a warrant for illegal practice was obtained for one ——— Rescigno, of 130 Dante Place, Buffalo, claiming to be a graduate of Naples, Italy, but not registered. He is endeavoring to get permission of the Regents to enter the next examination and to give him a chance, his case was put over in the city court until March 1, 1911.

The following named persons have been arrested for unlawful practice of medicine and their cases held for hearings as follows:

November 15, Henry F. Crane, of 437 Prospect Avenue, Buffalo, claims to have registered in Buffalo health office in 1885, and as a graduate of a vitopathic college of Cincinnati, Ohio, 1884, also of the American health college of Cincinnati. Not registered in County Clerk's office, law existing at that time, that of Chapter 513 of 1880, required presentation to county clerk of diploma and affidavit to register, claims diploma to have been burned. An examination of Cliny's list of medical colleges does not show such colleges as "vitopathic" or "American health" either in force or extinct among those in Ohio.

Crane's case was heard December 10 in the City Court; he plead guilty and on the recommendation of the chairman, in behalf of the medical society, in view of his advanced years and being a civil war veteran, and his promise to abstain from practising medicine, he was released on suspended sentence.

December 1, Fannie Ciresi, "Dr." on cards and sign, reading "Fannie Ciresi, M. T. D." of 315 Terrace, Buffalo, case set down for a hearing in city court, January 6, 1911. December 1, Elizabeth Fisher, of 522 Carlton Street, trance doctor, giving drugs, case set down for December 20, 1910. December 12, a warrant issued for one James Consatino of 366 Seneca Street, Buffalo, for attempt to treat a case of paralysis in which it is claimed the patient's thigh bone was broken.

The fine of $50 paid into city court by Ferdinand Schoenneman, of Hamburg, March 8, 1910, was paid October 31, 1910, to the chairman and by him turned over to the society's treasurer. Another fine of $50.00 recently paid by Martin Rybcynzki, rather than serve 50 days in jail has been claimed and will probably be adjusted within a short time.

During the year $500.00 has been assessed and paid into court by violators of the medical law, $400.00 of this has been paid to the society and there remains to be adjusted and paid $100.00.

For the first time in its history so far as can be learned, the board of censors has not had to call upon the treasurer for financial aid, except for moneys collected for fines.

A resumé of the Board's work shows for the year:

| | |
|---|---|
| Warrants issued for violations of the medical law | 19 |
| Arrested | 17 |
| Absconded | 2 |
| Convicted | 8 |
| Fined | 6 |
| Sentence suspended | 2 |
| Discharged | 7 |
| Awaiting trial | 4 |
| Special investigations of irregularities in medical practice | 2 |
| License to practice revoked | 1 |
| Undetermined | 1 |

Making a total of 20 cases, besides many other complaints have been investigated, the whole of them involving a great deal of work, time and patience. A financial report of the receipts and disbursements with vouchers pertaining thereto will be filed with the treasurer of the society at the end of the year.

Respectfully submitted,

John H. Grant
Francis E. Fronczak
DeLancey Rochester.

Dr. F. Park Lewis, chairman of the committee on legislation, presented his report in which he stated that, through a misunderstanding, the county and state societies were opposed to each other in certain legislation before the state legislature last year, and, therefore, offered resolutions, which were adopted, as follows:

WHEREAS, It is essential, if effective action is to be secured, that the legislative committee of the State medical society and the legislative committee of the various county medical societies act in concord in securing desirable and in opposing pernicious legislation,

*Resolved,* That the house of delegates of the State Medical Society be requested to devise some plan by which concurrence of action between these various county and state committees may be secured. Carried.

The chairman offered also the following:

WHEREAS, In many of the large cities, nearly 50 per cent. of the obstetricy is under the direction of unlicensed and untrained women, and

WHEREAS, There are no state regulations controlling the work of these women, be it, therefore,

*Resolved,* That the medical society of the county of Erie request the House of Delegates of the New York State Medical Society to direct the legislative committee of the state society to investigate existing conditions, and to report back to the society a draft of a law, for the licensing and control of midwives, for the consideration and approval of the state society.

Dr. Snow then offered the following:

*To the Governor and Legislature of the State of New York:*

A resolution of the medical society of the County of Erie requesting the health commissioner of the State of New York to undertake the field of investigation of epidemic poliomyelitis and also requesting the legislature of the State of New York to grant a sufficient sum of money to carry on the work.

Epidemic poliomyelitis or infantile paralysis has been for the last four years prevalent in the cities and agricultural districts of New York state, causing many deaths and hundreds of crippled children. The disease is known to be communicable both by direct contact and through healthy carriers. It is now present in nearly every part of the state, either in small epidemics or sporadic cases, each case being a focus of infection for the extension of the disease. For this reason, the state health commissioner has made poliomyelitis a reportable disease with a strict quarantine for several weeks.

The disease has a varied symptomatology, mild cases simulating grippe, neuritis, or rheumatism, severe cases resembling meningitis, all types being equally contagious requiring much experiience and knowledge to diagnosticate. Local health officers have jurisdiction only over their own limited territory and are unable to prevent the extension of the disease to adjoining towns. Experience has shown that country districts are most scourged with the disease.

The attention of the state health commissioner is called to the fact that poliomyelitis is prevalent in Canada, along the Niagara frontier; that there is constant intercourse during the summer between Canada and New York state, and that many cases of infantile paralysis in Buffalo in 1910, were probably infected in Canada, and that interchange of information and an agreement as to quarantine regarding poliomyelitis between Ontario and New York state would be most desirable.

The medical profession of Erie county believes a few cases of poliomyelitis may occur in the state during the cold season and that from these foci of contagion the disease will spread in a dangerous manner in the summer of 1911. It is obvious that the disease can only be watched and controlled by a strong central authority, the state health commissioner, and that he should have a staff of skilled specialists at his disposal. On request, the state health commissioner should be able to send a thoroughly qualified investigator to any part of the state to study the nature and extent of an epidemic of poliomyelitis and to issue instructions to prevent its spread. This is called the field investigation of the disease. The legislatures of Massachusetts, Pennsylvania, and several western states have appropriated generous sums of money to fight poliomyelitis, the children's plague. In view of all these facts, be it

*Resolved,* That the medical society of the county of Erie respectfully request the health commissioner of the state of New York to actively undertake the field investigation of poliomyelitis and request the legislature of New York State to appropriate sufficient money to carry on the work. Be it further

*Resolved,* That a copy of these resolutions be sent to Eugene H. Porter, health commissioner of New York, and if approved by him he be asked to forward the same to Governor John A. Dix, after he takes office, and the senate and assembly of the state of New York.

Passed at the annual meeting of the medical society of the county of Erie, December 19, 1910.

Dr. Wall made a point of order that this being legislative matter, it ought to go through the proper channels. The point of order was decided by the president as not well taken, and Dr. Snow's resolution was adopted.

Dr. Henry R. Hopkins, chairman of the committee on public health, then read the report of that committee:

WHEREAS, Ernest Wende departed this life on February 11, 1910, and

WHEREAS, Ernest Wende honored this society by every act of his life, and

WHEREAS, Ernest Wende served this society for many years with efficiency and inspiration as member and chairman of its Committee on Public Health; therefore be it

*Resolved,* That the Medical Society of the County of Erie hereby and herein declares and proclaims its high appreciation of the lofty character and the singular success of our lamented fellow member, Ernest Wende, as a man, as a member and officer of the society, and as a sanitarian and health commissioner.

*Resolved,* That this attempt of appreciation become a part of the records of the society, and a copy of the same be suitably engrossed, attested, and transmitted to the family of the deceased.

PETER W. VANPEYMA,
EDWARD CLARK,
HENRY REED HOPKINS, Chairman,
Committee on Public Health.

*Resolved,* That the Medical Society of the County of Erie heartily endorses the wise recommendation of Health Commissioner Wende of 1908, relating to the matter of defective school children and of open air schools.

*Resolved,* That we consider this subject to be one of supreme importance to Buffalo, by reason of the enormous number of such defectives; more than 8,000, and also by reason of the long well known poor ventilation in nearly all of our public schools.

*Resolved,* That we urge this matter upon the attention of the Department of Education, as one of vital importance and one which demands prompt attention and intelligent and efficient treatment.

PETER W. VANPEYMA,
EDWARD CLARK,
HENRY REED HOPKINS, Chairman,
Committee on Public Health.

WHEREAS, Buffalo has a school population of more than one hundred thousand of possible pupils, and

WHEREAS, the air supply of the rooms in which these children are taught is a factor of primal importance, as regards the preservation of health, the prevention of acute epidemic disease and also that there may be efficiency and economy in the conduct of our schools, and

WHEREAS, The records of our Board of Health demonstrate beyond question or cavil that the air of most of our public school rooms is polluted to a degree to constitute a distinct menace to the health and lives of our children, and

WHEREAS, The work of the medical inspectors of schools further demonstrates that from 10 to 17 per cent. of our school children are physically defective, and

WHEREAS, Our school records indicate that a large per cent. of pupils are known as "repeaters" the survey of this subject of

October, 1909, giving the number of such "repeaters" as 17,117; therefore

*Resolved,* That the attention of the several departments concerned in this matter—those in authority,—be invited to this important problem, to the end that suitable investigations be made; (a) to determine the degree of the ventilation of our school rooms; (b) to determine the physical defects, if any, of our "repeaters;" (c) to determine what responsibility, if any, the physical condition of our school rooms bear in causing the infirmities of our defective school children and in recruiting our repeaters.

*Resolved,* That the Medical Society of the County of Erie hereby records its conviction that this matter of the ventilation of our schools is a question of enormous significance,—economic, educational and social.

PETER W. VANPEYMA,
EDWARD CLARK,
HENRY REED HOPKINS, Chairman,
Committee on Public Health.

WHEREAS, The schools of Buffalo, public and parochial, represent in the main an investment for lots, buildings, and apparatus of over $9,000,000.00 and an annual cost for maintenance of some $2,000,000,00 and

WHEREAS, This enormous burden of our taxpayers is constantly increasing in both directions, and

WHEREAS, The provisions for the ventilation of these school buildings is the more important part of their construction both as to economy and efficiency of operation, and

WHEREAS, Since 1901, all plans for school buildings built by our Department of Public Works have contained most accurate, minute and intelligent provisions and specifications relating to the construction and testing of the ventilating apparatus of such buildings, and

WHEREAS, Persistent inquiry has so far failed to find the official records of the tests required by the plans and specifications of the many school buildings so planned and built since 1901, therefore

*Resolved,* That this important matter be brought to the attention of His Honor Mayor Fuhrmann, with the request that the subject matter receive his most careful consideration.

HENRY REED HOPKINS, Chairman.
EDWARD CLARK,
Committee on Public Health.

WHEREAS, The Medical Society of the County of Erie has frequently gone upon record in support of the proposition that the royal family, the people of the State of New York, deserved the services of a medical profession whose every member is fully prepared for the discharge of the high obligations and responsibilities of that profession, and

WHEREAS, It is the belief of this Society, that the preparedness required of those who would practise medicine in this State, is still much below that required of such candidates in many countries of Europe, or of candidates for admission into the medical service of the American Army or Navy, and

WHEREAS, The power to fix the standard of requirements for admission to the ranks of the medical profession of this State is vested in the Regents of the University and in its Board of Medical Examiners; therefore

*Resolved,* That the Medical Society of the County of Erie hereby requests the attention of the Medical Society of the State of New York to this important subject; and further suggests that the said State society take suitable steps to procure, if possible, the joint petition, of the several state medical societies, to the Regents of the University, asking in substance that the standard of admission to the right to practise medicine in the State of New York be raised and raised, until it can be said in truth and in fact, that there are none higher in the world.

HENRY REED HOPKINS, Chairman,
PETER W. VANPEYMA,
EDWARD CLARK,
Committee on Public Health.

Preamble: There are in the city of Buffalo, a large number of children of school age having physical and mental defects which unfit them for the usual class work provided. This is especially noticeable in children having defective eyes. In many instances vision is reduced to one-half or even one-quarter of the normal, as a result of early inflammation, leaving conditions which can never be bettered. Other children having developed myopia, if they are required to meet the usual school conditions become increasingly more near-sighted with continued use of the eyes.

It is desirable that children of these grades be taught under special conditions. It would also be possible for many who now go to the state school for the blind, having partial sight, to be trained in our public schools, if classes and teachers, for those having defective vision, were provided. This could be done at a great saving to the community and with advantage to those partially blind in that a certain proportion of them could be kept at home and thus saved the necessity of institutional training, therefore

*Resolved,* That the Medical Society of the County of Erie requests the Superintendent of Education to consider whether the arrangements already made are fully adequate for the reception of defective children, and whether special classes for those having imperfect eyes might not with great advantage be arranged.

HENRY REED HOPKINS, Chairman,
PETER W. VANPEYMA,
EDWARD CLARK,
Committee on Public Health.

The resolutions relative to the late Health Commissioner Wende were adopted by a unanimous rising vote. All the other resolutions were also adopted.

Relative to the last resolution, Dr. Blaauw said that defective ears as well as defective eyes require attention in the public schools. The committee answered, however, that it was deemed unwise to go too far, and would rather make a beginning with the eyes.

In the absence of H. J. Mulford, chairman of the committee on necrology, the report of committee was read by Dr. Getman.

The several memorials were unanimously adopted by rising vote.

C. Sumner Jones, chairman of the milk commission, presented his report as follows:

At a meeting of the Council of the Medical Society of the County of Erie, held April 4, 1910, President Wende, in accordance with the state law of 1908, appointed a milk commission for Erie County as follows:

Charles Cary, DeWitt H. Sherman, Norman K. MacLeod, bacteriologist and secretary, John Wende, veterinarian, and C. Sumner Jones, chairman. This action was approved by the society, at a regular meeting held April 18, 1910.

According to the report in 1909, of Otto P. Geier, Secretary of the American Association of Medical Milk Commissions, there are some fifty-eight milk commissions, and these, through an interchange of ideas, established certain certified milk standards. The same authority also informs us that three states—New York, Kentucky and New Jersey,—give legal sanction to the term "certified."

The history of legally certified milk in Erie county is still in its infancy. The commission is not, as yet, highly, technically, organized. Nor has the field of certified milk production in Erie county been invaded by but few enterprising producers. Erie county was one of the last of the counties of the three states, that have thus far complied with the legal enactment relative to the sale of certified milk.

It has been the effort of this commission to put into active effect, only such requirements of the producer, as seemed best suited and necessary to protect the consumer against the sale of infected, impure and harmful milk. Numerous other like commissions have published reports of their methods, and from these reports your commission has been able to glean much valuable information to apply in its work.

Thus far, the owners of three dairies have made application for, and have received permission to sell their product as "certified milk." The first certification was made on July 7, 1910, to the dairy of Mr. S. M. Clement, of East Aurora, and the Queen City Dairy Company of this city was given authority to sell this product. The daily sale, at first, was about fifty quarts, and now the Queen City Company report a daily sale of one hundred and eighty quarts.

On July 9, certification was made to the goat dairy of Mrs. James C. McKeand, 159 East Tremaine Avenue, Kenmore, N. Y. The product of this dairy is small, ranging up to eight or ten quarts daily. Messrs. Smither and Thurston, of 279 Bryant Street, have authority to sell the same as certified goats milk.

The third application was from Mr. E. M. Holmes, of Yorkshire, N. Y., proprietor of the "Fairholme Dairy." On August 26, the commission certified to this dairy, and the product of same is being sold by J. B. Soper, 1148 West Avenue. The quantity sold is about three hundred and twelve quarts daily.

Certified cows milk is being sold in Buffalo, at twelve cents a quart, and certified goats milk at fifty cents a quart. A prospective company is now being organised with the view of applying for authority to sell certified milk in Buffalo.

The following is the general form of the letter which the commission has addressed to the applicant, where the commission has certified to a dairy.

Mr............................

.....................N. Y.

Dear Sir—This is to certify that the Milk Commission of the Medical Society of the County of Erie, N. Y., has inspected your farm and dairy, .................N. Y., and has caused your herd to be tested. The veterinarian reports that an examination and tuberculin test has been made of the milking cows, and that he considers them free from any contagious and infectious disease. We are satisfied that the barn, feed, drainage, and water supply, and other provisions are satisfactory. Our bacteriologist pronounces the milk to be free absolutely from antiseptics, or added preservatives, and from bacteria in excessive numbers, thus meeting the requirements for certified milk as laid down by the American Milk Commission. We shall continue making tests, and in the meantime, you are authorized to advertise and sell the product of your dairy as "Certified Milk," bearing the certification and marked with the name of this commission. We reserve the right to withdraw this authority for cause.

MEMBERS OF THE COMMISSION. Yours truly,

.............................. THE MILK COMMISSION OF
.............................. THE MEDICAL SOCIETY OF
.............................. THE COUNTY OF ERIE, BUF-
.............................. FALO, N. Y.
..............................

..................Chairman.

As a practical working basis, the commission has followed, in general, the requirements as adopted by the milk commission of the Academy of Medicine of Toronto, Canada.

Bacteriological and Chemical—There must be no pus, blood, or injurious germs. A maximum average bacterial count of 10,000 is allowed during June, July, August and September, and it is expected that an average of not to exceed 5,000 will be maintained during the other months. The fat content should be 4 per cent., but a variation of 5 per cent. above or below, is permitted. The total solids should not fall below 12 per cent.

The milk must neither be watered nor frozen, nor must any preservative be added. The milk should be cooled to 45° F. within half an hour after milking, and kept between 40° and 45° till delivered to the consumer. It must be delivered within twenty-four hours after milking.

The veterinarian requires that the tuberculin test be applied to all cows before admission to the herd, and to the entire herd twice yearly afterwards. We exclude suspicious as well as typical reactors. He is also to report as to the cleanliness of the dairy, and the cleanliness and apparent health of the employees, and to any matter of a hygienic nature.

Report of the bacteriologist average monthly counts shows as follows; each c. c. of milk:

| | |
|---|---|
| Mr. Clement's dairy (sold by Queen City Dairy Co.) | 5,984 |
| Mrs. McKeand's goat dairy | 10,000 |
| Fairholme dairy | 6,788 |

Queen City Dairy Co's own report by months, shows:

July 6,400, August 8,900, September 8,500, October 7,930, November 5,600, December 3,300.

No provision appears to have been made to defray the necessary expenses of the milk commission, and the commission would respectfully recommend that a committee be appointed by the County Medical Society to provide ways and means for meeting this expense, if in your judgment such provision is desirable. At present, the bacteriologist and the veterinarian render their charges directly to the producer.

The commission invites the council of this society to act as an advisory board in making any suggestions that may facilitate the usefulness of the commission.

The time should be near at hand when only milk conforming to the standards of certified milk shall be permitted to be sold under any name. It is also to be hoped that through the potent teaching of the medical profession in general, and the individual physician in the home, the public mind may soon come to realize more fully the great importance of clean milk as a food.

Respectfully submitted,
C. SUMNER JONES, *Chairman.*

The forgoing report was received and on motion, the recommendations contained therein were approved.

Dr. J. D. Bonnar, chariman of the committee on contagious diseases hospital, reported for that committee as follows:

STATUS OF HOSPITAL FOR CONTAGIOUS DISEASES.

When many remedies are prescribed for a disease, there is obviously lack of knowledge, either of its true nature or of the proper action of the remedies used. That the diagnosis of the urgent necessity for a hospital for the most modern treatment of patients afflicted with such contagious diseases as scarlatina, measles, diphtheria and others of less virulence, was correctly made is proved by the prompt and almost spontaneous response of all parts of the city, through its various civic organizations, of men and women alike, the culmination of this popular approval being the equally prompt and willing approval by our municipal government in granting the appropriation of $150,000 for the construction of the hospital plant required. Thus, was shown the correctness of the diagnosis made by the medical societies, county and academy, in originating this humanitarian movement, some six months previous to the successful passage of the measure.

That the remedy for the complaint has thus far proven deficent in curative properties, need not be discussed before this audience, yet its lessons may well enlist your thoughtful attention for a few moments. I am assured, from close familiarity with the incidents of this work, that the medical profession is a unit in sympathy and personal support of this sanitary project, which is alike favored by a united popular will. It is also conceded by medical men and students of sanitary science, that the local influence would rather lessen than increase the spread of such diseases. Yet, for reasons not warranted by strict statistical proof (of the immunity of neighbors to such diseases, when they are cared for in hospitals of modern design), we find one section of the city after another rebelling against such neighbor.

The Best street and Dewey Avenue sites were opposed by popular prejudice, while other locations have been named as available, yet not wholly acceptable to allied interests. The Bailey and Kensington avenues site, while possessing many physical qualities of highly commendable worth, has not the approval of your committee, or rather of the joint committee of the county society and academy of medicine, who went out there last July, I think it was, to inspect its features. While admiring its physical features, its long distance from the center of medical service, rendered it impracticable. This, at least, was the concensus of opinion. The Hamlin site was then being advocated by the chamber of commerce for a city hospital, which sought to unite the county hospital and hospital for communicable diseases in one grand institution. Our committees, while not enlisted in that worthy work, were, however, in sympathy therewith.

Its efforts, thus far, are not decisive, yet warrant our interest and co-operation, passively or actively, as seems wisest at each stage of its progress. The joint committee of the Aldermanic and Councilmanic bodies has taken up the question with the Supervisors as to which would build the hospital, to be denominated The City Hospital. Its report, which I will read and file with this society as part of my own, upon this occasion, has just been given me today, upon its presentation to the board of Aldermen, which received it this afternoon. The action of both houses thereon is awaited with much interest by your committee. That the solution is coming nearer seems logical, even if not visible.

Nothing is ever settled till it is settled right, so, while perplexed with the seeming inconsistencies of delay, yet the crucible of public necessity and public sympathy in this humanitarian need must and inevitably will work out the remedy, and so effectually dispel and remove the dross of prejudice, that united effort will supplant opposition and the disheartening delay of the present, will finally be rewarded by such ampler institution than first designed, our city will lead in this co-operative mission of mercy. Your hospital committee always ready, when the door of opportunity is open, for its effectual work, awaits such, but is willing and receptive, at all times, of practical suggestions.

JOHN D. BONNAR, M.D., *Chairman.*

Report was received and committee continued.

Dr. John H. Pryor, Chairman of the Committee on "Division of Fees," presented the report of that committee, and then moved its acceptance and the adoption of the recommendations contained therein. Dr. Wall moved, as an amendment, that the committee be continued, the amendment to the motion being carried. The motion as amended was then adopted.

Dr. Grosvenor called the attention of the society to the proposed amendment to the constitution of the state society which calls for active and associate membership. After explaining the question, Dr. Grosvenor moved that our delegates to the state society be instructed to oppose this amendment to the constitution. Carried.

Dr. Wall moved that a committee be appointed to consider the question of the consolidation of the various medical organisations into one body. Carried.

Dr. Wall presented the following report:

As your representative on the committee to arrange for a joint meeting of our society and the Academy of Medicine, the Pharmacal Society and the Chemical Society, to be addressed by Dr. H. W. Wiley, of Washington, D. C., I beg to report that the meeting will be held on the evening of January 31, 1911, at the Y. M. C. A. Hall, and Dr. R. Park will preside.

Dr. Wiley will be met at the depot Tuesday morning, taken to the University Club for breakfast; then taken to the Falls, returning for supper at the Club, in time to arrive at the meeting at 8 p. m. The morning of February 1 will be devoted to showing him points of interest about the city, and he leaves at 1 p. m., on the Empire Express. A reception committee of four from each society, together with the committee of arrangements, are appointed to meet him and accompany him, so far as convenient; the expense is to be divided among the four societies. I ask your approval and authority to make all necessary arrangements to carry out the program.

Committee members from this society: Drs. Wende, Cohen, Park, Hurd, and Wall. Our share of the expense will not be over $35.00.

The report was received and authority asked for granted.

The report of the tellers was then called for by the president. Dr. Francis, for the tellers reported as follows:

Votes cast for president—Dr. Cohen—62

Votes cast for president—Dr. McClure—62

There being a tie for the office of president, the chair suspended action on this office until the report on the others had been heard and acted upon. Tellers then reported that for first vice-president, Dr. McKee had received 83 votes and Dr. Bayliss 11 votes.

The president declared Dr. McKee elected first vice-president.

For second vice-president, Dr. Helwig received 68 votes, Dr. A. R. Gibson, 12 votes, and Dr. Helwig was declared duly elected second vice-president.

For secretary, Dr. Gram received 68 votes and Dr. Edmund Reiman 26 votes. The chair declared Dr. Gram duly elected secretary.

For treasurer, Dr. Lytle received 69 votes and Dr. Frisch 21. Dr. Lytle was declared duly elected treasurer.

For censors, the votes were cast as follows:

John H. Grant 71; A. G. Bennett 64; F. E. Fronczak 61; L. Hendee 59; G. L. Brown 53; Battaglia 28. The first five named having received the highest number of votes were declared duly elected censors.

For chairman of the committee on legislation, Dr. F. Park Lewis received 60 votes and Dr. O'Gorman 10. The chair declared Dr. Lewis duly elected.

For chairman of the committee on public health, Dr. H. R. Hopkins received 51 votes and Dr. A. C. Schaefer 22. Dr. Hopkins was declared duly elected.

For chairman of the committee on membership, Dr. C. A. Wall received 50 votes and Dr. Preiss 15. Dr. Wall was declared duly elected.

For delegates to the Medical Society of the State of New York, the following five were declared elected, with these votes:

J. H. Pryor 57; Frederick Busch 61; Edward Clark 60; G. J. Eckel 45; Eli H. Long 53.

The chair then took up the question of the tie vote for president. Dr. Francis, for the tellers, stated that they had only had time to go over the vote once and would like very much to have a new committee appointed to canvass the ballots for president again.

Dr. Wall then moved that the chair appoint three tellers to recount the ballots for president. Carried.

The president appointed, as such committee, Drs. Wall, Bennett and Schaefer. They immediately recounted the ballots in public, calling the name written on each ballot, so that anyone desirous of doing so could keep tally.

The result of the recount showed that Dr. McClure had received 65 votes and Dr. Cohen 61 votes. The chair then declared Dr. McClure duly elected to the office of president for the ensuing year, and introduced him to the society. Dr. McClure briefly thanked the society for the distinguished honor conferred upon him, whereupon the meeting adjourned.

---

